# Exploring reproductive trajectories of youths of Oromia, Ethiopia: A life course approach

**DOI:** 10.1371/journal.pone.0279773

**Published:** 2022-12-30

**Authors:** Tariku Dejene, Eshetu Gurmu

**Affiliations:** Center for Population Studies, College of Development Studies, Addis Ababa University, Addis Ababa, Ethiopia; Johns Hopkins University Bloomberg School of Public Health, UNITED STATES

## Abstract

In the context of continuous cultural, social, and economic changes happening around the globe, the predictable patterns of the life course of the past observed over successive birth cohorts will not remain stable across generations. In this study, three reproductive role indicators—first sexual encounter, first marriage, and first birth–for three synthetic birth cohorts were used to identify and characterize the reproductive trajectories of youths. In our analysis, for the sake of comparison with global literature, we considered youths to be between ages 15 and 24. The analysis was conducted using data extracted from the 2005, 2011, and 2016 Ethiopian Demographic Health Survey for Oromia National Regional State. Three synthetic birth cohorts of youths of birth years between 1975 and 1989 were constructed for the analysis. A sequence analysis based on dynamic hamming distance with partition around medoids technique was employed to extract the typologies of reproductive trajectories of youths. In addition, discrepancy analysis and a sequence regression tree analysis were employed to characterize the identified typologies of trajectories. Data management was done using STATA 14 and all analyses were carried out using R software. The study identified four different typologies of reproductive trajectories among the youth. The sex of respondents was the primary discriminating factor of the typologies of reproductive trajectories. The findings support the notion of changing norms in reproductive behavior among the less educated youth irrespective of sex. The discriminating power of education was stronger for female youth in urban areas than rural females. It implies that the postponement of reproductive role assumption was stronger among educated female youths residing in urban than their rural counterparts. Normative reproductive practices such as early marriage and adolescent fertility are still common practices that require efforts of communities and local government bodies to ameliorate these practices. Results of the study indicate that less educated youth should be targeted in programs that aim at improving youth empowerment (i.e., training and employment opportunities) as well as their sexual and reproductive health.

## Introduction

Based on biological and psychological characteristics, a clear distinction can be made between children and adults. Children progress through stages in their life course and eventually assume adult roles. Although children are perceived to be apolitical and unengaged in economics, this image of innocence will not be held in the case of youths [1]. Youth is a stage in the life course of an individual where the process of transitioning from childhood to adulthood happens. The transition period is a time of change in which young people make choices about their future and respond to these changes [2, 3]. Thus, youth is a critical stage in human development during which a young person leaves childhood behind and assumes new roles and responsibilities. In our analysis, youth is defined as groups of people who are between the ages of 15 and 24.

The journey that young people take to adulthood involves transitions in many aspects of their lives. Here, rather than a single event, boundary crossing to adulthood is defined by participation in a variety of activities. These activities include a range of socioeconomic and demographic events. Finishing school, entering the labor force, becoming financially secure, getting married, and becoming a parent are some of the major events commonly used as markers of transition to adulthood [3–5]. Multiple processes, such as productive and reproductive, occur in the lives of individuals in such a way that the processes themselves interact with each other [6, 7].

Ethiopia has been undergoing demographic, social, and political transformation since the 1960s. The country had experienced political upheaval that led to regime changes during the previous century. For instance, the end of the imperial era in 1974 ushered in a period of political and social change in Ethiopian history [8–10]. Two decades later, the country adopted a formal population policy that recognized the interdependence among population, resources, the environment, and development [11]. In the MDG era, between 2000 and 2010, several policies and strategies regarding adolescent and youth reproductive health were introduced. These policies and strategies aimed to regulate and govern family relations and improve access and utilization of reproductive services [12–14].

Over a long time period, dramatic changes occurred in Ethiopia in terms of access and enrollment in primary and secondary education. Enrollment and gender parity in primary school, for example, have both improved. Furthermore, as a result of the introduction of free primary schooling as a policy intervention, the dropout rate has slowed down [15]. With the changes happening in the area of education and urbanization, young people have acquired increased agency that enabled them to make decisions at the moment than the previous generation regarding their reproductive behavior [16]. In this study, we attempted to investigate the reproductive trajectories of youths using debut to sex, entry to marriage, and parenthood as markers of reproductive transition in the context of a changing policy environment and productive roles of youths.

The timing, duration, and sequence of life events have been studied using the life course approach in the context of a changing social and economic environment [17–19]. By situating individual and family development in cultural and historical contexts, the theory seeks to discern the various factors that shape people’s lives from birth to death [20]. This perspective extends the limitations of researches that focus on single transition event by viewing life course as a unit composed of a series of diverse status transitions over time. This theory was used in this study to evaluate the hypothesis that the interaction of individual life and historical time, known as the cohort effect, shapes the reproductive trajectories of youths. In the current study, we attempted to explore the reproductive trajectories of three birth cohorts of youths born between 1975 and 1989. Each of these birth cohorts represents a group that grew up in a different historical period and policy context, which may have influenced their lives. The table below (Table 1) summarizes the birth cohorts as well as the historical context in which they grew up [11–14, 21, 22].

**Table 1:** Definition of birth cohorts and historical contexts, Ethiopia.

Birth Cohorts	Birth year	Year Age 15	Year Age 25	Historical and Policy Context
Cohort 1	1975–1979	1990–1994	2000–2004	1991 End of Civil War 1993 National Population Policy
Cohort 2	1980–1984	1995–1999	2005–2009	1993 National Population Policy 1997 Health Sector Development Program I 2000 Revised Family Code 2002 Health Sector Development Program II 2003 Health Extension Program 2004 National Youth Policy
Cohort 3	1985–1989	2000–2004	2010–2015	1993 National Population Policy 2000 Revised Family Code 2002 Health Sector Development Program II 2003 Health Extension Program 2004 National Youth Policy 2006 National Adolescent and Youth Reproductive Health Strategy 2007–2015

## Methods

The data for the study was extracted from Ethiopian Demographic and Health Surveys (EDHS). The EDHS sample was designed to provide estimates of key demographic and health variables for the entire country, urban and rural areas separately, and each of Ethiopia’s nine regions and two city administrations separately. Residence stratified and two-stage cluster sampling technique was used to collect the data. Enumeration areas (EAs) from urban and rural areas were initially chosen randomly. In stage two selection, a full listing of the selected EAs’ households was conducted. In the second round of the selection process, a predetermined number of households per EA were chosen from the list of households [23–25].

Synthetic birth cohorts for birth years 1975–1979 from the 2005 survey, 1980–1984 from the 2011 survey, and 1985–1989 from the 2016 survey were constructed. The reproductive experiences of these three birth cohorts of youth were investigated using debut to sex, entry into marital union, and parenthood as indicators of reproductive transition. The reproductive trajectory of youths was constructed by ordering the timing of debut to sex, age at first marriage, and age at first birth in their life course before the age of 25 years. Thus, the reproductive trajectory is made up of four potential categorical states. The initial state for all observations is *single*, that is, never had sex, not married, and didn’t start having children. Youths may stay at this initial state or advance to either *premarital sex* or *marriage*. The final potential state is becoming a *parent* before the age of 25 or otherwise.

The complexity of the trajectories arises from the sequencing of the states, the timing, and their duration of stay in the various states. The sequence data was prepared only for ages starting from age 15 to 24. Potential demographic and socio-economic determinants of reproductive trajectories were also extracted. The list included sex (1 = male and 2 = female), educational attainment at the time of the survey (1 = no education, 2 = primary, and 3 = secondary and above), place of residence (1 = urban and 2 = rural), and household wealth (1 = lower and 2 = higher).

Sequence analysis is best suited to investigate the occurrence and timing of events, as well as the duration between events, by focusing on the trajectories rather than the occurrence of a single event. The analysis uses a range of algorithms that attempt to quantify dissimilarities between life-course trajectories. The algorithms compute pairwise dissimilarities between sequences and then use these dissimilarities to identify trajectory typologies by clustering the sequences [26–28]. While some measures are highly sensitive to sequencing, some others are exceedingly sensitive to timing, and the remainders emphasize the importance of spell duration [28]. For this study, the choice of type of algorithm was dictated by the fact that sensitivity to the timing of life events is more highly relevant than sensitivity to ordering or spell lengths.

A Dynamic Hamming Distance (DHD), a measure highly sensitive to the timing of life events in sequence analysis, was used to compute the dissimilarities. These are then used to group similar trajectories using a partition around medoids (PAM) algorithm. When compared to hierarchical clustering, PAM has the advantage of maximizing a global criterion rather than just a local criterion. In addition, the choice of an optimal number of partitions and quality assessment of the partitions were made through the aid of an Average Silhouette Width (ASW) and Point Biserial Correlation (PBC) measures [29].

After the groups have been identified, an assessment of the association between clusters of trajectories and factors such as birth cohort, sex, place of residence, educational attainment, and household wealth was made using a multifactor discrepancy analysis. Discrepancy analysis computes a covariate’s contribution to the dissimilarity in the reproductive trajectory. Thus, it allows for the ranking of factors based on their relative importance in explaining the disparity in reproductive trajectory. A regression tree method was also utilized to discover the most significant discriminant covariates and their interaction [30].

The regression tree-growing process starts with all individuals being put in one node and recursive partitioning based only on significant factors will be done. During the partitioning, the predictor and the split are chosen in such a way that the resulting nodes differ as much as possible from one another [30]. This recursive process was repeated until the last partition step captures a minimum of 5% of the total number of weighted cases. Split significance was assessed at a p-value of 5% for an F-test with five thousand permutations.

The data management, data editing, and data preparation for the analysis were done using STATA 14.0 [31]. The analysis, however, was carried out in R software version 4.2.0 [32, 33] using *TraMineR* and *WeightedCluster* packages. *TraMineR* package was used to analyze and visualize state sequences including the multifactor discrepancy analysis and sequence regression tree; whereas, the *WeightedCluster* package was used to partition sequence dissimilarity and assess the quality of the partition [29, 34]. The analysis was carried out using weighted cases.

The study was conducted based on secondary data obtained from the Demographic and Health Surveys Program. Procedures and questionnaires for standard Demographic and Health Surveys have been reviewed and approved by the ICF/ORC Institutional Review Board. In addition, the study protocols obtained ethical clearance from the Institutional Review Board offices of the Ministry of Science and Technology of Ethiopia and the Ethiopian Health and Nutrition Research Institute. Interviews were conducted after securing oral consent from respondents. Furthermore, the names of respondents and individual identifiers were not included in the final data to ensure respondents’ anonymity.

## Results

The first birth cohort (births from 1975–1979) constituted 1083 respondents, 1775 respondents were used for the second birth cohort (births from 1980–1984), and the last birth cohort considered (births from 1985–1989) was made up of 1753 respondents. Rural respondents made up a considerable share of the samples throughout all birth cohorts: 86.3% of the first birth cohort, 85.9% of the second, and 88.4% of the third birth cohort samples were from rural areas. Nearly three-quarters of the samples of the first birth cohort (74.4%) were female youths. This gap in percentage was a result of the difference in sampling strategy followed for male and female samples during the 2005 survey. In the second and third birth cohorts, female youths accounted for 56.3% and 57.2% of the sample (Table 2).

**Table 2:** Background characteristics of respondents, Oromia–Ethiopia.

Birth Cohorts	Sex (%)		Residence (%)		Total (Number)
	Male	Female	Urban	Rural	
1975–1979	25.6	74.4	13.7	86.3	1083
1980–1984	43.7	56.3	14.1	85.9	1775
1985–1989	42.8	57.2	11.6	88.4	1753

**Fig 1:**
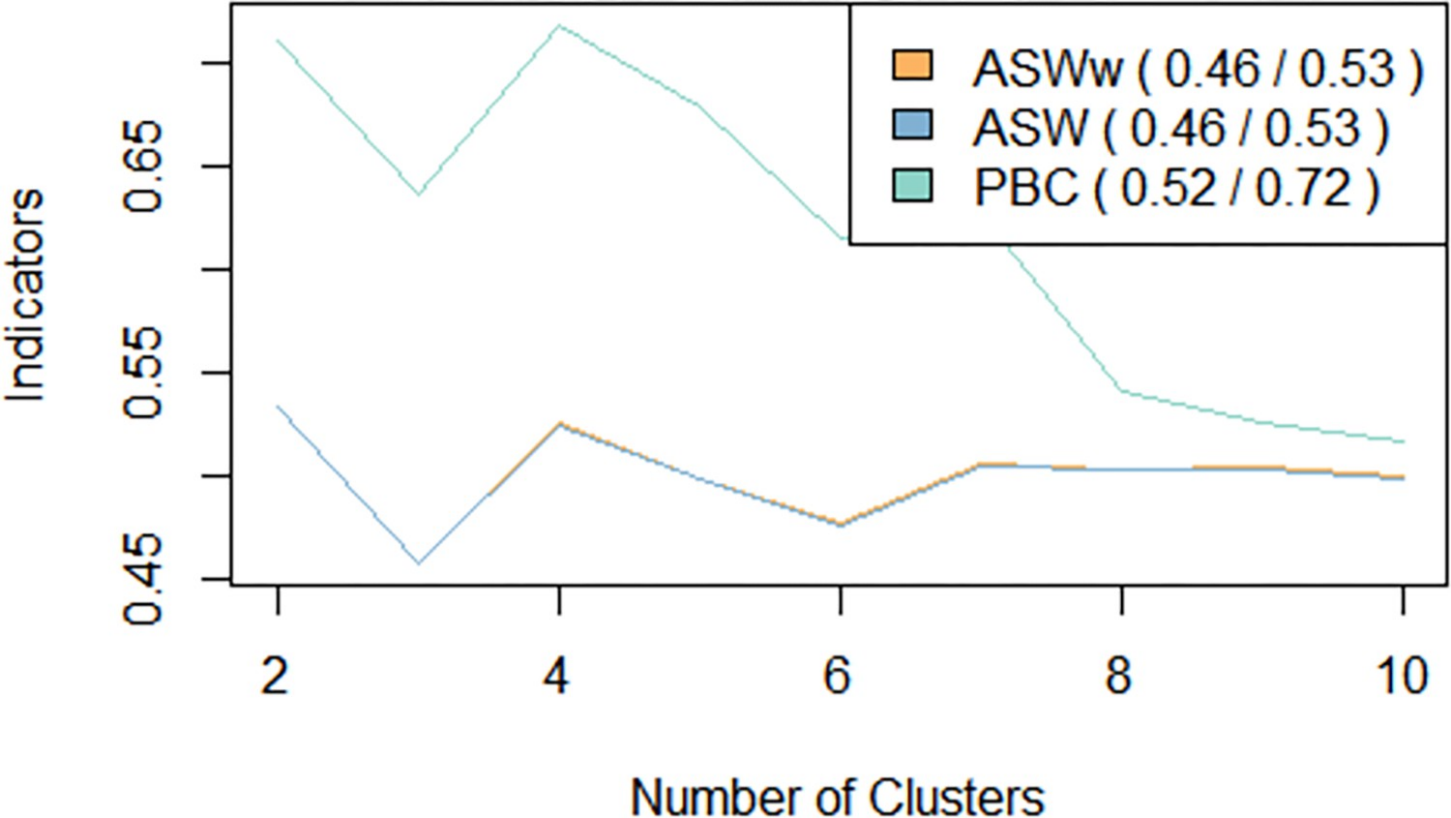
Choosing an optimal number of clusters. (ASW = Average Silhouette Width, ASWw = Average Silhouette Width (weighted), PBC = Point Biserial Correlation).

**Fig 2:**
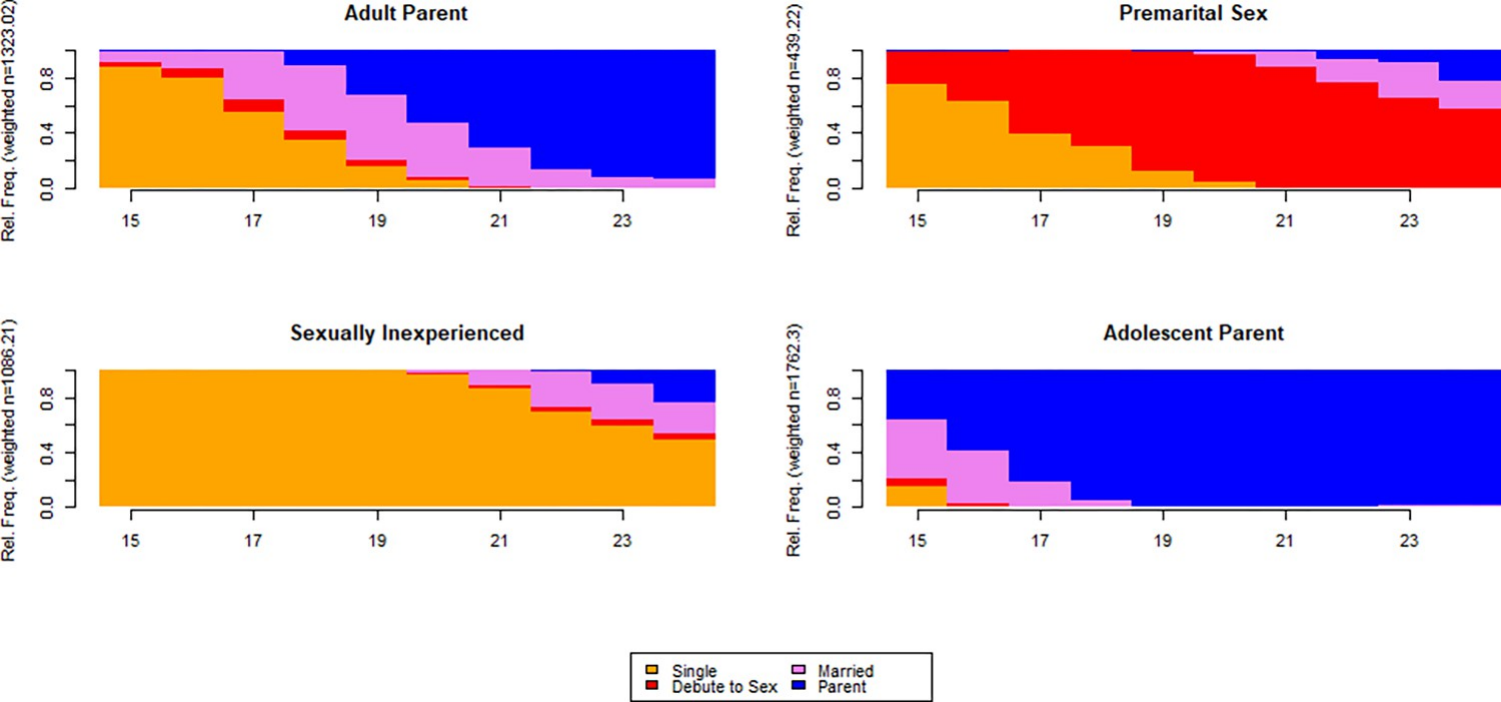
Typology of youths reproductive trajectories, Oromia–Ethiopia.

The analysis identified four types of reproductive trajectory typologies for youths of Oromia. The partitioning around medoids (PAM) algorithm was used to identify these typologies. A variety of quality assessment indicators were used to evaluate the partition’s quality. An average silhouette width (ASW = 0.53) and its weighted version (ASWw = 0.53) statistics suggested a partition of two typologies that somewhat oversimplified the pattern of reproductive transition to adulthood. However, the point biserial correlation (PBC = 0.72 and ASW = 0.52) suggested a solution of four typologies as appropriate, yielding diverse pathways of youth’s reproductive trajectories. The ASW values suggest the presence of an underlying reasonable structure (Fig 1). The typologies are depicted using a chronogram plot (Fig 2) and descriptions of the typologies are provided hereunder.

1. **Sexually inexperienced**: These groups of youths exhibited a typical characteristic of abstaining from sex, marriage, and having their first child before 24 years of age. In terms of magnitude, this typology ranks third constituting 1086 of the respondents (23.6% of total respondents of all birth cohorts). A small proportion of these youths marry after the age of 20, and the likelihood of transitioning from marriage to having a child was slimmer as sex before marriage is considered taboo in major parts of Ethiopia.
2. **Adolescent parent:** This typology ranks first in magnitude encompassing 1762 youth respondents of all birth cohorts (38.2%). Youths of this cluster characteristically get married too early and become a parent. Nearly all of the youths of this group got married by age 16 and become a parent by age 18.
3. **Adult Parent**: This group accounted for 1323 youths of all birth cohorts (28.7%) making the cluster the second most popular typology of youth’s reproductive pathway to adulthood. Contrary to the characteristic of an adolescent parent, these groups of youths on average postpone marriage until around age 18. Further, for this cluster, the transition from marriage to the birth of the first child takes an average of two years.
4. **Premarital sex**: This is the least prevalent typology of youth’s reproductive transition to adulthood accounting for 9.5% of respondents of all birth cohorts. Two characteristic features define this group of youths; *i*) transition to marriage and parenthood before the age of 24 was less likely and *ii*) youths’ debut to sex begins before age 18.

The multifactor discrepancy analysis revealed that the reproductive trajectory that youths assume in their life course before age 25 was shaped by multiple factors, each of which contributes differently to the overall pattern of reproductive transition trajectory. The global F statistics and R-squared value provide that the model explains a significant proportion of the trajectory discrepancy (F = 51.7, R^2^ = 0.174, and p-value = 0.000). As can be seen from the table below (Table 3), socio-economic status, as measured by household wealth, didn’t have an independent significance in explaining the discrepancy in the reproductive trajectory of youths (R^2^ = 0.001 and p-value = 0.133). The sex of respondents was the most significant factor that shaped the discrepancy in reproductive trajectory having the largest share of R-square value (R^2^ = 0.099 and p-value = 0.000). This significant portion of the discrepancy in reproductive trajectory demonstrates the presence of gender disparity in terms of the reproductive transition of youth. In addition to sex, in order of their relative importance in explaining the discrepancy of the reproductive trajectory of youths, we found the level of education of youths (R^2^ = 0.011 and p-value = 0.000), birth cohort (R^2^ = 0.004 and p-value = 0.001), and place of residence (R^2^ = 0.002 and p-value = 0.005).

**Table 3:** Multifactor discrepancy analysis of reproductive trajectory, Oromia–Ethiopia.

**Variables**	**Pseudo F**	**Change in R** ^**2**^	**P-value**
Sex	205.7	0.099	0.000
Education	11.5	0.011	0.000
Birth cohort	3.9	0.004	0.001
Residence	3.5	0.002	0.005
Wealth	1.6	0.001	0.133
	**Pseudo F**	**R** ^**2**^	**P-value**
Global	51.7	0.174	0.000

**Fig 3:**
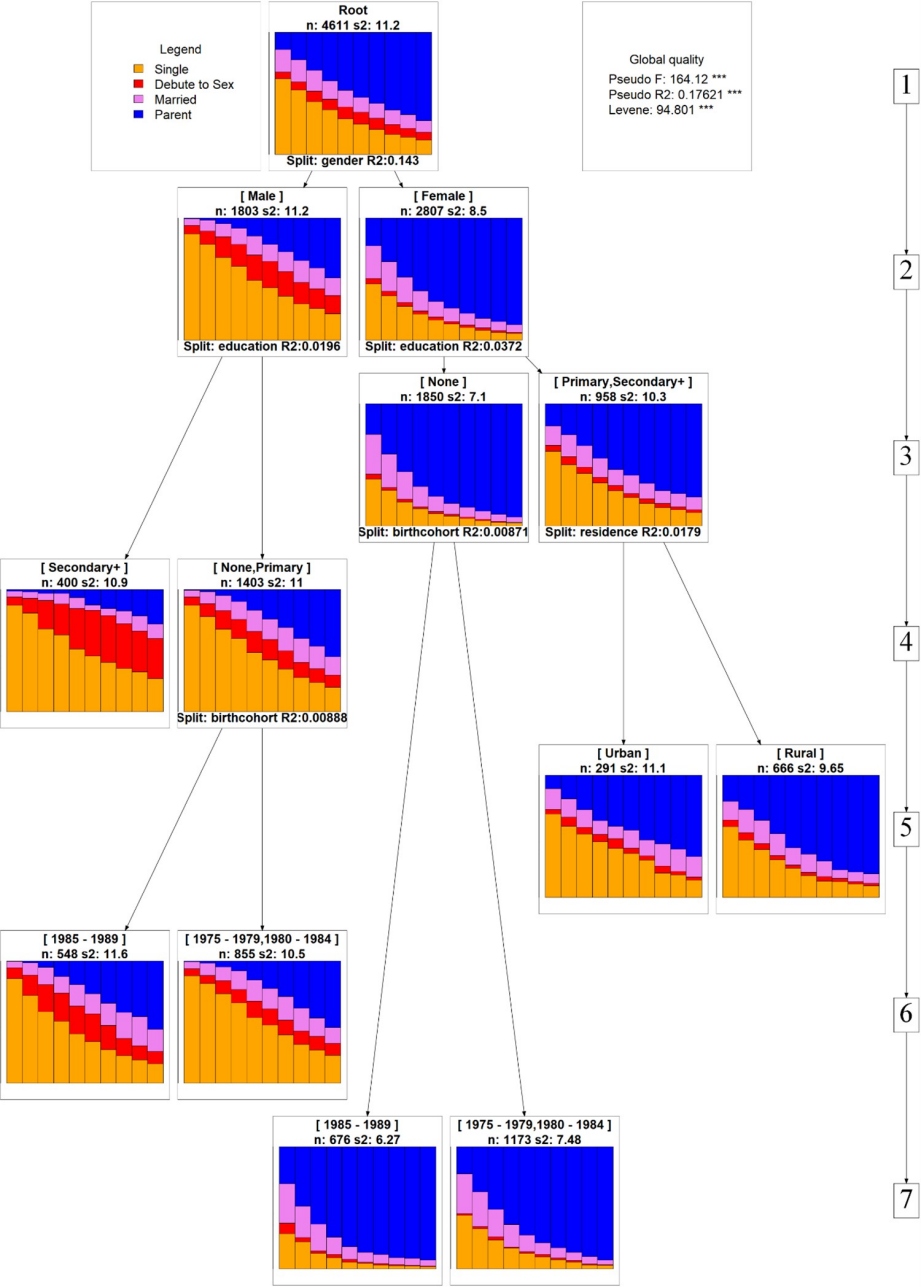
Sequence regression tree, Oromia–Ethiopia.

Although the multifactor discrepancy analysis permitted an assessment of the effect of factors on the discrepancy of the reproductive trajectory, it didn’t allow us to evaluate how the trajectories get modified as these factors change. A sequence regression tree analysis result portrays not only the effect on the discrepancy of the trajectory but also the interaction of covariates that weren’t accounted for in the multifactor discrepancy analysis. The results indicate that the regression tree explained 17.6% of the total discrepancy observed showing that the evolution of the reproductive trajectories of youths in the life course significantly differed between groups. The global Pseudo-F and Levene’s tests also attested to the fact that the trajectories varied significantly more between groups than within groups (Fig 3).

Sex was the first covariate to significantly contribute to the between group discrepancy in trajectory, accounting for 14.3% of the total between group discrepancy. While female youths transition to marriage and parenthood before the age of 24 with a relatively higher magnitude as compared to male youths, premarital sex was a characteristic feature that identified the trajectory of male youths. The second most significant factor that contributed to the discrepancy in trajectory in both male (R^2^ = 1.96%) and female (R^2^ = 3.72%) youths was education. The results portray that those female youths with no formal schooling transition to marriage and parenthood the earliest than those with at least a primary level of schooling. Additionally, male youths with secondary and above levels of schooling were much more likely to engage in premarital sex and delay getting married and having children until later in life. Contrarily, premarital sex is a rare occurrence among male youths with no formal education or only a primary level of education (Fig 3).

Another intriguing finding from the regression tree analysis was that the pattern of the trajectory did not remain stable across birth cohorts among male youths who did not complete primary school (R^2^ = 0.89%). Transition to sex, marriage, and parenthood happened earlier for the recent birth cohorts of males (1985–1989) than in previous birth cohorts. Regardless of its small size contribution to the discrepancy (R^2^ = 0.87%), birth cohorts differentiated the trajectory of female youths without formal schooling. Transition to marriage and parenthood occurred at an earlier age for females of recent birth cohorts (1985–1989) having no formal schooling as compared to females of older birth cohorts (1975–1979 and 1980–1984) without formal schooling (Fig 3).

Place of residence was also found to be a discriminating factor but the discriminating power was significantly higher among educated females contributing to a 1.79% of trajectory discrepancy. Unlike this, the benefit of educational attainment of female youths in postponing reproductive roles to later in life was not evenly distributed among urban and rural residents. The benefit of female education, primary and above level, in pushing reproductive roles to later in life was better translated in urban areas than the rural settings (Fig 3).

## Discussion

The study aimed at identifying underlying reproductive transition patterns of youths in Oromia National Regional State of Ethiopia. In addition, the sources of the diversity of the reproductive life trajectory of youths were also investigated. Instead of focusing on a single transition event, the research considered life course as a unit composed of a series of diverse status transitions over time. Thus, the analyses were made using three reproductive role indicators: debut to sexual intercourse, first marriage, and first birth. These indicators were selected as these events are known to have a far-reaching impact on an individual’s health and well-being in adulthood [35].

This study has identified four typologies of the reproductive trajectories of youths. The first one is a transition to family formation and parenthood early in adolescent life which was the most prevalent typology. The second most common typology was characterized by deferring marriage and parenthood until age 18 but before 20. The third and fourth typologies account for one-third of the youths having common characteristics of postponing marriage and parenthood beyond age 24. However, youths in the third cluster were found to refrain from sex, marriage, and parenthood before the age of 24 while those who belong to the last cluster were characterized by postponing marriage and parenthood to later ages although engaged in sexual activity at a young age.

### Traditional norms and values are still dominating youths’ reproductive trajectories

Numerous research outputs reported the emergence of multiple reproductive trajectories with heterogeneous patterns in various locations [17–19, 36, 37]. The results of this study add to the body of youth reproductive health research demonstrating the existence of diverse reproductive trajectories in the study setting. The findings indicate that a large number of youths engage in the traditional type of reproductive role; they marry and have children at an early age. However, the identified patterns of reproductive behaviors also demonstrate that youth’s reproductive behavior was diverging from the tradition of getting married and having children early.

In our analysis, an effort was made to map not only the continuation but also the patterns of divergence of reproductive practices from the normative types of roles. Consistent with our findings, others have also reported both the continuation and discontinuity of these traditional reproductive practices. The discontinuation was a result of a variety of factors such as the expansion of formal education, better employment opportunities, and urbanization [38–41]. The findings strongly suggest that male and female youths’ reproductive trajectories differed substantially. In line with this, earlier studies have shown that, in comparison to male youths, female youths typically transitioned to marriage and took on parental roles early in their lives [25, 42]. However, this pattern did not remain stable and varied across different characteristics of youths indicating the presence of a strong interaction effect with the sex of youths.

### Education appears to affect the sexual and reproductive behavior of young persons in Ethiopia

Education is a transformative experience that increases youths’ awareness of alternative roles. As a result of increased earnings after schooling, youths will be encouraged to postpone reproductive roles until later in life in the long run. For female youths, a primary level of schooling was an important divide having a significant impact in delaying marriage and childbearing. Conversely, female youths without formal schooling assumed a traditional reproductive trajectory. This is not a novel discovery in and of itself, as it has been noted that female education tends to delay the age of first marriage leading to a delay in age at first birth for females. In this regard, female secondary education had an even stronger association with delayed entry to marriage and parenthood than primary education [35, 43, 44]. A rather intriguing finding reported in this work was the fact that the postponement of reproductive role assumption was stronger among educated female youths living in urban areas than their rural counterpart. Female urban residents are more likely than rural ones to continue to secondary school [25]. The delayed entry to first marriage and childbearing of urban female youths compared to rural females could be attributed to the better progression of urban females’ to post-primary education. In the same vein, it is possible to argue that rural female youths’ early transition to reproductive roles set the stage for their poor progression to post-primary education.

### Gender disparity in reproductive trajectories

Male youths characteristically delay marriage and parenthood to later ages than female youths. Yet, the postponement of these reproductive roles to later life resulted in an increased rate of entry to premarital sexual activity. The findings support previous researches that reported male youths are more likely to defer their transition to marriage and parenthood than females [42, 45]. In the cultural context of the study area and generally in Africa, fatherhood entails ensuring the wellbeing of the family through financial provision; thus, fathers have an obligation to fulfill the needs of both their children and the mother of their children [45–47]. Delaying marriage and fatherhood may not necessarily be linked to fertility decisions of male youths to have fewer children; rather, it might be a behavior induced by resource constraints and higher unemployment rates male youths are experiencing [45, 48, 49].

Male youths with secondary and above levels of education defer marriage and parenthood to an even advanced age while engaging themselves in premarital sexual activity. Thus, first sex, marriage, and fatherhood were unlikely to be closely tied for these groups of male youths. A number of reasons such as peer pressure, personal sexual desire, exposure to media, and culture were cited in literature as causes for engaging in premarital sex among adolescents and youths [50–53]. Worryingly, the practices are likely to be unsafe leading to unplanned pregnancy, premarital fertility, abortion, and sexually transmitted infections (STIs) including HIV/AIDS.

The results suggested that younger birth cohorts of male youths with less than a secondary level of education transition to marriage and parenthood sooner than older birth cohorts. Similarly, among female youths with no formal schooling, the recent birth cohorts were disadvantaged in that the transition to reproductive role assumption occurred earlier than in older birth cohorts. Despite this, the changing sexual behavior and reproductive transitions across generations irrespective of sex have also been documented elsewhere [54]. Early transition to marriage and parenthood observed among the less educated younger birth cohorts of both sexes could potentially be a result of an increasing prevalence of premarital sex and unplanned pregnancy. Findings from Tanzania corroborate the fact that the early transition of less educated females to reproductive roles was commonly practiced in response to the increased risk of premarital sex [55].

#### Strengths and limitations

Finally, it is worth mentioning the strengths and limitations of the present analysis. The identification of different types of trajectories was made possible by taking into account the timing, spacing, and sequencing of multiple reproductive events all at once is the major strength of this study. Another unique contribution of the present study is the assessment of the interaction of effects of variables through the application of multifactor discrepancy analysis and sequence regression tree analysis. Further, the study was conducted for both male and female youths and enabled gender comparison of reproductive trajectories. Nonetheless, it should be noted that the data used for this study was gathered through retrospective reporting of events such as age at first sexual debut, age at first marriage or union, and age at first birth. The accuracy of data is dependent on the capacity of the respondents to remember the timing of the occurrences of the events that could be affected by a memory lapse. Thus, the results should cautiously be interpreted as data quality of these events might be affected by different types of reporting errors [56]. Further, our analysis was focused only on major covariates that discriminated the various reproductive trajectories observed and did not consider an exhaustive list of covariates.

### Implications for research and practical applications

Given the limitation of the present work, we recommend future researchers depart from the traditional binary classification of reproductive experiences and endeavor to explore reproductive trajectories through the lens of a holistic approach. Further, because sex accounts for a significantly larger portion of the diversity in reproductive trajectory, it follows that the possible reasons for the gender gap in the transition to family formation and parenthood trajectories require further investigation. Although education was found to be one of the trajectory discriminating factors, for rural resident female youths, the nature of the relationship between secondary and above levels of educational attainment and transition to reproductive roles was not clear.

Normative reproductive practices such as early marriage and adolescent fertility are still common practices (38.2%) that require efforts of the communities and local government bodies to ameliorate these practices. Although there are improvements scored in this regard as a result of various interventions, premarital sex, and its consequences are observed as worrisome emerging reproductive patterns among youths. This emerging pattern in a reproductive pattern is especially prevalent among the less educated males and females of the younger birth cohort implying that lack of access to education among this group is still affecting some aspects of their life course. They should be targeted in a program that aims at improving youths’ empowerment specifically by offering training and employment opportunities as well as widening their access to sexual and reproductive health services.

## Conclusions

The study identified four different typologies of reproductive trajectories among youths. The sex of youths was the primary discriminating factor of the typologies of reproductive trajectories. Female youths transition to marriage and parenthood earlier than males; for male youths, however, first sex, marriage, and fatherhood were unlikely to be closely linked. The study also showed that the reproductive trajectories of the three birth cohorts who lived in different cumulative historical time and policy environments exhibited slight but significant disparity. The findings support the notion of changing norms in reproductive behavior among the less educated male and female youths. Primary level educational attainment was a major factor in discriminating female youths into those who entered marriage and parenthood the earliest and those who postponed marriage and parenthood a little longer. The discriminating power of education was even stronger for female youths in urban areas than those living in rural settings.

## Supporting information

S1 Appendix IRB letters. (ZIP)

## Abbreviations

ASW: Average Silhouette Width
ASWw: Average Silhouette Width (weighted)
DHD: Dynamic Hamming Distance
EA: Enumeration Area
EDHS: Ethiopian Demographic and Health Survey
PAM: Partition around Medoids
PBC: Point Biserial Correlation
