# Supplementary material for: Exploring reproductive trajectories of youths of Oromia, Ethiopia: A life course approach
Source: PLoS One. 2022 Dec 30;17(12):e0279773. doi: 10.1371/journal.pone.0279773 (PMC9803128; doi:10.1371/journal.pone.0279773)
Supplement: S1 Appendix — (ZIP) [file pone.0279773.s001.zip › Approval Findings Form 2016 Ethiopia DHS.pdf]

Institutional Review Board Findings Form
ICF IRB FWA00000845 (exp. 04/13/2019)

Project Director(s): Yodit Bekele

Project Title: Ethiopia Demographic and Health Survey

ICF Project Number: 132989.0.000.ET.DHS.01

Type of Review:

☒ New ☐ Modification ☐ Annual review

Findings of the Board:

☒ Project complies with all of the requirements of 45 CFR 46, "Protection of Human Subjects"

☐ Project is exempt from IRB review (See IRB Exemption Form)

☐ Project does not comply with all of the requirements of 45 CFR 46

Project Approved Until: June 2017

Next Annual Review Date: October 15, 2016

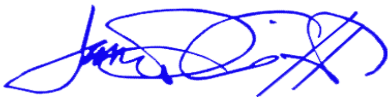

Chair, Institutional Review Board

October 15, 2015

Date

(Revised 07/18/2014)

List of Approved Project Materials:

1. 2016 EDHS Household Questionnaire
2. 2016 EDHS Woman's Questionnaire
3. 2016 EDHS Man's Questionnaire
4. 2016 EDHS Biomarker Questionnaire
5. 2016 EDHS Health Facility Questionnaire
6. 2016 EDHS Fieldworker Questionnaire
