# Supplementary material for: Exploring reproductive trajectories of youths of Oromia, Ethiopia: A life course approach
Source: PLoS One. 2022 Dec 30;17(12):e0279773. doi: 10.1371/journal.pone.0279773 (PMC9803128; doi:10.1371/journal.pone.0279773)
Supplement: S1 Appendix — (ZIP) [file pone.0279773.s001.zip › DHS-MOST-Approval May 2016.pdf]

በኢትዮጵያ ፌዴራላዊ ዴሞክራሲያዊ ሪፐብሊክ
የሳይንስና ቴክኖሎጂ ሚኒስቴር
The Federal Democratic Republic of Ethiopia
Ministry of Science and Technology

ቁጥር 3/10/114/2016
Ref. No.
ቀን May 9, 2016
Date

To: Ethiopian Central Statistics Agency

Addis Ababa

Re: 2016 Ethiopian Demographic Survey

Dear Sir/Madam//Mr./Mrs./Dr,

The National Research Ethics Review Committee (NRERC) has reviewed the aforementioned project protocol in an expedited manner. We are writing to advise you that NRERC has granted

Full Approval

To the above named project, for a period of **one year (May 9, 2016- May 8, 2017).** All your most recently submitted documents have been approved for use in this study. The study should comply with the standard international and national scientific and ethical guidelines. Any change to the approved protocol or consent material must be reviewed and approved through the amendment process prior to its implementation. In addition, any adverse or unanticipated events should be reported within 24-48 hours to the NRERC. Please ensure that you submit biannual progress report once in six months and annual renewal application 30 days prior to the expiry date.

We, therefore, request you as PI and your esteemed organization to ensure the commencement and conduct of the study accordingly and wish for the successful completion of the project.

With regards,

Yohannes Sitotaw
Secretary of NRERC

CC. _ **Ethiopian Public Health Institute (PI)**
_ **Mr. Asalfew Abera (PI)**
_ **NRERC Chairperson**

ማነጋገር ቢያስፈልግዎ
You may Contact

ፖ.ሳ.ቁ.
P.O.Box 2490

አዲስ አበባ ኢትዮጵያ
Addis Ababa, Ethiopia
E-mail most@ethionet.et

ስልክ
Tel. 251-011-4-674353
Web site:-http://www.most.gov.et

ፋክስ
Fax +251-011-4-66 02 41
