# Supplementary material for: Exploring reproductive trajectories of youths of Oromia, Ethiopia: A life course approach
Source: PLoS One. 2022 Dec 30;17(12):e0279773. doi: 10.1371/journal.pone.0279773 (PMC9803128; doi:10.1371/journal.pone.0279773)
Supplement: S1 Appendix — (ZIP) [file pone.0279773.s001.zip › Approval IRB Review Findings Form EthiopiaDHS2011.pdf]

ICF Macro
an ICF International Company

ICF Macro
Institutional Review Board

IRB Review Findings Form

Name of Project Director(s): Ann Way

Title of Project: 2010 Ethiopia Demographic and Health Survey

ICF Macro Project Number: 31561.00.042.00

Type of Review:

_X_ New

____ Renewal

Findings of the Board:

____ Project is exempt from IRB review

_X_ Project complies with all of the requirements of 45 CFR 46, "Protection of Human Subjects"

____ Project does not comply with all of the requirements of 45 CFR 46

Project Approved Until: February 28, 2011

Next Annual Review Date: September 11, 2010

[signature]
Chair, Institutional Review Board

September 11, 2009
Date

(Revised 04/08/09)

11785 Beltsville Drive, Suite 300 — Calverton, MD 20705 — 301.572.0200 — 301.572.0999 fax — icfmacro.com
