# Supplementary material for: Exploring reproductive trajectories of youths of Oromia, Ethiopia: A life course approach
Source: PLoS One. 2022 Dec 30;17(12):e0279773. doi: 10.1371/journal.pone.0279773 (PMC9803128; doi:10.1371/journal.pone.0279773)
Supplement: S1 Appendix — (ZIP) [file pone.0279773.s001.zip › EHNRI approval page EthiopiaDHS2011.pdf]

ETHIOPIAN HEALTH AND NUTRITION RESEARCH INSTITUTE(EHNRI)
SCIENTIFIC AND ETHICAL REVIEW OFFICE (SERO)
SCIENTIFIC AND ETHICAL REVIEW COMMITTEE (SERC)

PROJECT APPROVAL FORM

PROJECT No. **EHNRI/ SERO/SERC/03/2002**

PROJECT TITLE

2010 Ethiopian Demographic and Health Survey

COMMENT OF SERC

The above entitled project has been reviewed and was found to be scientifically valid and ethically clear. The outcome of the project is expected to provide evidence based information on population, health and nutrition at national level.

DECISION

APPROVED [X] NOT APPROVED [ ]

SIGNATURE OF THE SERC MEMBERS

Name	Signature
1. Dr Eshetu Lemma	[signature]
2. Dr. Belete Tegbaru	[signature]
3. Dr Getachew Addis	[signature]
4. Dr. Aseged Woldu	[signature]
5. Melke Tadesse	[signature]
6. Abebe Bekele	[signature]
7.	
8.	

Date of Approval Sept. 30 20[illegible]

[stamp: Federal Democratic Republic of Ethiopia, Ethiopian Health & Nutrition [illegible] Institute]

Sept. 30, 2009
