# Supplementary material for: Exploring reproductive trajectories of youths of Oromia, Ethiopia: A life course approach
Source: PLoS One. 2022 Dec 30;17(12):e0279773. doi: 10.1371/journal.pone.0279773 (PMC9803128; doi:10.1371/journal.pone.0279773)
Supplement: S1 Appendix — (ZIP) [file pone.0279773.s001.zip › DHS5 Approval.pdf]

Macro International Inc.
Headquarters
11785 Beltsville Drive
Calverton, MD 20705

Phone: 301-572-0200
Fax: 301-572-0999
www.orcmacro.com

ORC MACRO℠

ORC MACRO
Institutional Review Board

IRB Review Findings Form

Name of Principal Investigator(s): Martin Vaessen

Title of Project: Demographic and Health Surveys

Macro Project Number: 31406.00.002.12

Type of Review:

X New

____ Renewal

Findings of the Board:

____ Project is exempt from IRB review

X Project complies with all of the requirements of 45 CFR 46, "Protection of Human Subjects"

____ Project does not comply with all of the requirements of 45 CFR 46

Project Approved Until: September 30, 2008

Next Annual Review Date: July 13, 2006

John A. [illegible] (signature)
Chair, Institutional Review Board

08/30/05
Date
